# Brain Metastases From Solid Cancers in 58 Dogs

**DOI:** 10.1002/vms3.70441

**Published:** 2025-06-02

**Authors:** Samuel Okonji, Federica Rossi, Silvia Sabattini, Massimo Baroni, Federica Poli, Riccardo Zaccone, Simone Perfetti, Gualtiero Gandini, Laura Marconato

**Affiliations:** ^1^ Department of Veterinary Medical Sciences Alma Mater Studiorum University of Bologna Bologna Italy; ^2^ Anicura Clinica Veterinaria dell'Orologio Bologna Italy; ^3^ Clinica Veterinaria Valdinievole, Pistoia Monsummano Terme Italy

**Keywords:** canine, cerebral, haemangiosarcoma, metastatic

## Abstract

Brain metastases (BM) represent an unmet medical need in human medicine, and they are poorly documented in dogs. The aim of this multi‐centre retrospective study was to report the clinical characteristics, primary solid cancer histology, advanced imaging findings, treatment modalities and potential prognostic factors in dogs with presumed BM that occurred either at the time of initial diagnosis or during follow‐up. BM diagnosis was established through either imaging studies or histologic examination of specimens obtained during necropsy. A total of 58 client‐owned dogs with histologically proven solid cancer and BM were included. Clinicopathologic variables, BM characteristics based on imaging and survival post‐BM (SPBM) were recorded. Haemangiosarcoma (53.4%) and carcinoma (27.6%) were the most common primary tumour histotypes, followed by melanoma (12.1%) and undifferentiated sarcoma (6.9%). Synchronous BM and solitary BM occurred in 63.8% and 51.7% of dogs, respectively. The prosencephalus was most commonly affected, with 79% of dogs showing neurologic deficits. Antitumoural or palliative treatment was attempted in a minority of dogs, with no improved outcome. The median SPBM was 3 days (range, 1–255). The 3‐ and 6‐month survival rates were 8.6% and 1.7%, respectively. Dogs with haemangiosarcoma (OR: 7.6; 95% CI, 2.2–25.8; *p* = 0.001) and those with distant metastases at presentation (OR: 16; 95% CI, 4.2–60.9; *p *< 0.001) had an increased likelihood of developing synchronous BM. Haemangiosarcoma and carcinoma were the tumours most frequently associated with BM, which were more commonly synchronous and symptomatic, with a high incidence of forebrain localization. The prognosis was poor, regardless of the primary cancer type.

## Graphical abstract

Brain metastases (BM) are poorly documented in dogs. The aim of this study was to report the clinical characteristics, primary solid cancer histology, advanced imaging findings, treatment modalities and potential prognostic factors in dogs with BM. A total of 58 dogs with histologically proven solid cancer and BM were included. Haemangiosarcoma and carcinoma were the tumours most frequently associated with BM, which were more commonly synchronous and symptomatic, with a high incidence of forebrain localization. The prognosis was poor, regardless of the primary cancer type.

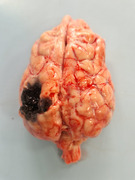

## 1 Introduction

Metastasis is the leading cause of cancer‐related death worldwide, representing the most demanding challenge in modern medicine (Seyfried and Huysentruyt 2013).

In people, the incidence of brain metastases (BM) has increased, largely due to an ageing population, the advent of targeted therapies that have extended the survival of cancer patients and improvement in early cancer detection (Singh et al. 2022; Kamar and Posner 2010). Approximately 20% of human cancer patients are reported to develop BM; however, necropsy studies suggest the true incidence may reach up to 40% (Nayak et al. 2012; Tabouret et al. 2012; Tsukada et al. 1983). Patients with lung cancer (40%–50%), breast cancer (15%–25%) and melanoma (5%–20%) have the highest prevalence (Singh et al. 2022).

BM are often associated with severe neurologic and cognitive impairment, along with disappointing survival rates. Computed tomography (CT) is typically performed in the acute setting for rapid intracranial assessment and detection of potential neurosurgical emergencies (Pope 2018). CT is also a useful tool to detect haemorrhage, calcifications, and evaluate osseous structures (J. R. Fink et al. 2015). However, magnetic resonance imaging (MRI) remains the preferred modality for BM detection (Kaufmann et al. 2020). This imaging technique provides excellent soft tissue contrast, allowing for detailed and high‐resolution visualization of brain anatomy (Kaufmann et al. 2020). BM pose a treatment dilemma due to the poor permeability of the blood‐brain barrier to systemic therapy and their frequent occurrence in advanced‐stage disease, indicating significant spread beyond the primary site (Kamar and Posner 2010).

Historically, patients with BM have a poor prognosis, with survival typically measured in weeks if left untreated and months with a multidisciplinary approach (Singh et al. 2022; Kamar and Posner 2010).

Brain metastases also occur in dogs, but their true incidence is difficult to estimate due to limited data, mostly derived from older studies predating the widespread use of advanced imaging. Many of these studies rely on autopsy reports that significantly underrepresent the actual number of cases (Song et al. 2013; Kishimoto et al. 2020). Overall, their prevalence is considered low, ranging from 0.2% to 2.2%, with haemangiosarcoma, metastatic carcinomas of various primaries and melanoma historically accounting for the majority of BM (Song et al. 2013; Kishimoto et al. 2020; Snyder et al. 2006). While this trend may reflect the general prevalence of these tumours, certain subgroups of primary malignancies may have a higher biological propensity towards metastatic spread to the brain, as documented in human patients (Nayak et al. 2012; Habbous et al. 2020). The rate of BM may also vary according to primary tumour size, presence of nodal or distant metastases and histologic malignancy (Habbous et al. 2020; Waqar et al. 2018; Koniali et al. 2020).

The aim of this retrospective study was to report the clinical characteristics, primary non‐lymphomatous solid cancer histology, advanced imaging findings, treatment modalities and potential prognostic factors in dogs with presumed BM that occurred either at the time of initial diagnosis or during follow‐up.

## 2 Materials and Methods

The study did not fall within the application areas of the Italian legislative decree 26/2014, which governs the protection of animals used for scientific or educational purposes; therefore, ethical approval was waived for this study. Dogs were treated according to the current standards. All owners provided written informed consent for the use of their animals' data for scientific purposes.

Electronic medical records of three Italian referral veterinary clinics were retrospectively reviewed to identify dogs diagnosed with histologically proven non‐lymphomatous solid cancer and suspected BM over a 13‐year period (2011–2024), either at the time of initial diagnosis or during follow‐up. All consecutive cases of BM were retrieved by one author for two referral centres, while another author gathered cases from the third centre. Search terms included ‘metastases’, ‘metastatic’, ‘brain’, ‘cerebral’, ‘cerebellar’ and ‘central nervous system’.

Cases were ultimately included if they were diagnosed with BM using one of the following modalities:

1. Total‐body computed tomography (TBCT) performed for staging purposes of a primary cancer. Both dogs undergoing TBCT for staging a tumour at its initial presentation and those undergoing TBCT at a later stage due to progression of the known cancer were included. A total of 5 min delayed brain scan was added to the standard protocol if a brain lesion was suspected in the first post‐contrast series. BM was diagnosed in the presence of round/oval single or multiple cerebral or cerebellar, as previously described (Hecht and Adams 2010; Wisner et al. 2011; Wisner and Zwingenberger 2015; Kraft and Gavin 1999), or brain stem space‐occupying lesions with variable enhancement, occurring in combination with primary extracerebral cancer, and potentially associated with other sites of metastatic spread.
2. Brain MRI performed on animals with primary extracerebral cancer presenting with clinical signs of intracranial disease. BM were diagnosed based on the presence of intra‐axial rounded to oval space‐occupying lesions, which appeared isointense to hypointense on T1‐weighted images (T1W) and hyperintense on T2‐weighted images (T2W), showing homogeneous, heterogeneous or peripheral contrast enhancement. Additional features supporting the diagnosis of BM were the presence of perilesional oedema and a predilection for the grey–white matter junction and vascular border zones (Hecht and Adams 2010). Haemangiosarcoma BM was suspected in cases showing a heterogeneous appearance, particularly when susceptibility effects were present on GET2*‐weighted images due to intratumoural haemorrhage (Wisner et al. 2011; Wisner and Zwingenberger 2015). If suspected BMs were identified in dogs initially undergoing brain MRI, additional imaging was conducted to determine the primary origin of the tumour.
3. Necropsy performed on dogs with solid cancer, either with or without neurologic signs.

Overall, dogs with single brain lesions were included if necropsy and histopathology confirmed the metastatic origin, if TBCT revealed concurrent multifocal visceral or nodal secondary lesions, or if MRI features suggested metastatic disease in the presence of an extracerebral solid tumour (Hecht and Adams 2010; Wisner et al. 2011).

Dogs were excluded if an extracranial primary cancer was not identified or if they had multiple synchronous primaries (i.e., two or more different malignant tumour types).

Regardless of the method of identification, we chose an arbitrary threshold of 30 days to define the timeframes for classifying BM. Synchronous BM were defined as those detected during the initial diagnosis of the primary neoplasm, while metachronous BM were those identified more than 30 days after the primary cancer diagnosis.

The following clinical and pathologic variables were analysed: signalment (breed, age, sex, weight), primary cancer histotype, date of initial diagnosis of primary cancer, date of BM diagnosis, number, size and location of BM, patterns on TBCT or MRI, neurologic signs, presence/absence and location of extracranial metastases, treatment for BM (none vs. symptomatic vs. antitumoural), date of progression of BM, date and cause of death. The availability of all the above information was not mandatory for inclusion.

All TBCT and MRI studies were reviewed by four authors (two European College of Veterinary Neurology residents, one experienced European College of Veterinary Neurology diplomate, and one experienced European College of Veterinary Diagnostic Imaging diplomate) to identify morphologic TBCT or MRI patterns consistent with BM (Bentley 2015; Mallol et al. 2022; K. R. Fink and Fink 2013). All necropsy studies were conducted by a board‐certified veterinary pathologist.

The presence of neurologic signs was gathered from clinical records and was determined through neurologic examination or, in the case of epileptic seizures, from anamnestic details provided by the owners. Survival data were obtained from the medical records and, if required, through telephone interviews with the owners or referring veterinarians.

### 2.1 Statistical Analysis

Categorical variables were summarized as frequency (percentage), while numerical variables were summarized as median (range).

Binary logistic regression was applied to assess the influence of potential prognostic variables on the likelihood of developing synchronous BM. Evaluated variables included purebred, sex, age, weight, primary cancer location, tumour histotype, presence of nodal metastases at first presentation and at BM onset, presence of distant metastases at first presentation and at BM onset, number of BM (solitary or multiple lesions), BM longest diameter (≤ or > 1 cm), and BM location (prosencephalon, brainstem, cerebellum).

Differences in BM characteristics, including lesion number, size and location, among the main tumour histotypes (haemangiosarcoma, carcinoma, melanoma) were also evaluated using binary logistic regression. The effect size of the variables was expressed as odds ratios (ORs) with their corresponding 95% confidence interval (CI).

Survival post‐BM (SPBM) was defined as the duration, in days, from the date of BM diagnosis or the onset of neurologic signs or the dogs with BM diagnosed post‐mortem to either the date of death or the closure of data collection. Dogs deceased from any cause were considered as events. Death due to tumour‐related causes included cases where death or euthanasia was attributed to the primary tumour, extracranial metastases, and/or BM, as it was not always possible to determine the predominant cause.

The influence of the above variables on SPBM was evaluated through univariable and multivariable Cox proportional hazards regression analysis. Continuous variables such as age and body weight were dichotomized using the median value as the cut‐off point. Covariates that exhibited a significant *p* value in univariable tests were subsequently incorporated into a multivariable regression model. Data were analysed with SPSS v.26 (SPSS, Inc., IBM, Chicago, IL, USA). *p* ≤ 0.05 were considered significant.

## 3 Results

### 3.1 Dogs' and Tumours' Characteristics

The database search revealed 78 dogs with presumed BM, all of which were assessed for eligibility. A total of 12 dogs were excluded due to the absence of histologic confirmation of the primary tumour and 8 because imaging was limited to the head only.

A total of 58 dogs met the inclusion criteria. There were 45 (77.6%) pure‐breed dogs and 13 (22.4%) mixed‐breed dogs. The most represented breed was the Boxer (*n* = 7; 12.1%), followed by the German Shepherd (*n* = 4; 6.9%), whereas 20 breeds were represented once or twice. A total of 35 (60.3%) dogs were males (10 neutered), and 23 (39.7%) were females (14 neutered). The median age was 11 years (range, 6–16), and the median weight was 27.6 kg (range, 3.7–46.0). The most common primary cancer histotypes were haemangiosarcoma (*n* = 31; 53.4%) and carcinoma (*n* = 16; 27.6%), followed by melanoma (*n* = 7; 12.1%), undifferentiated sarcoma (*n* = 3; 5.2%) and stromal sarcoma (*n* = 1; 1.7%).

Overall, BM were diagnosed at initial presentation in 37 (63.8%) dogs (synchronous metastases), while in the remaining 21 (36.2%) dogs, BM were diagnosed during the course of the disease (metachronous metastases) after a median time of 339 days (53–1021) from initial primary cancer diagnosis. Dogs and tumour features are listed in Table 1.

**TABLE 1:** Main demographic and clinical findings observed in a cohort of dogs with brain metastasis, stratified by primary tumour histotype.

	Haemangiosarcoma (*n *= 31)	Carcinoma (*n *= 16)	Melanoma (*n *= 7)
Age (median [range])	11 (7–14) years	11 (6–16) years	11 (10–15) years
Sex			
Male	20 (64.5%)	8 (50.0%)	5 (71.4%)
Female	11 (35.5%)	8 (50.0%)	2 (28.6%)
Primary tumour location	spleen (*n* = 11), heart (*n* = 8), skin/subcutis (*n* = 3), liver (*n* = 2), lung (*n* = 2), retroperitoneum (*n* = 2), muscle (*n* = 1), kidney (*n* = 1), bone (*n* = 1)	mammary gland (*n* = 4), lung (*n* = 4), nasal cavity (*n* = 1), skin (*n* = 1), liver (*n* = 1), anal sac (*n* = 1), prostate (*n* = 1), kidney (*n* = 1), digit (*n* = 1), thyroid gland (*n* = 1)	oral (*n* = 5), digit (*n* = 2)
Nodal metastasis at presentation	1 (3.2%)	7 (43.8%)	2 (28.6%)
Distant metastasis at presentation	27 (87.1%)	7 (43.8%)	3 (42.9%)
Timing of BM			
Synchronous	26 (83.9%)	8 (50%)	0 (0.0%)
Metachronous	5 (16.1%)	8 (50%)	4 (100.0%)
Presence of neurologic signs	17/23 (73.9%)	10/13 (76.9%)	6 (85.7%)
BM location			
Prosencephalon	29 (93.5%)	16 (100.0%)	6 (85.7%)
Brainstem	4 (12.9%)	0 (0.0%)	0 (0.0%)
Cerebellum	6 (19.4%)	3 (18.8%))	1 (14.3%)
BM maximum diameter (median [range])	13 (2–40) mm	15 (8–26) mm	11 (6–30) mm
Number of BM			
Single	11 (35.5%)	11 (68.8%)	7 (100.0%)
2–10	17 (54.8%)	5 (31.2%)	0 (0.0%)
> 10	3 (9.7%)	0 (0.0%)	0 (0.0%)
Nodal metastasis at BM diagnosis	5 (16.1%)	10 (62.5%)	1 (14.3%)
Distant metastasis at BM diagnosis	29 (93.5%)	11 (68.8%)	5 (71.4%)

### 3.2 Dogs With Haemangiosarcoma

Regarding the 31 dogs with haemangiosarcoma, the presence of BM was documented through TBCT in 20 (64.5%), MRI in 5 (16.1%), and necropsy in 6 (19.4%) dogs. The necropsy was also performed on two of the dogs diagnosed via TBCT and on one diagnosed through MRI. In all these nine cases undergoing necropsy, the diagnosis of BM from haemangiosarcoma was confirmed.

The primary tumour was localized in the spleen (*n* = 11, 35.5%), heart (*n* = 8, 25.8%), skin/subcutis (*n* = 3, 9.6%), liver (*n* = 2, 6.5%), lung (*n* = 2, 6.5%), retroperitoneum (*n* = 2, 6.5%), muscle (*n* = 1, 3.2%), kidney (*n* = 1, 3.2%) and bone (*n* = 1, 3.2%).

At the initial diagnosis, 26 (83.9%) dogs had synchronous BM, and the remaining 5 (16.1%) developed metachronous BM after a median time of 240 days (range, 142–392). At the time of BM diagnosis, all but two dogs had either distant (*n* = 24; 77.4%) or nodal and distant (*n* = 5; 16.1%) metastases.

A total of 11 (35.5%) dogs had a single brain lesion, while in the remaining dogs, the median number of BM was 7 (range, 2–100). Lesions were located in the prosencephalon (*n* = 22, 70.9%), prosencephalon and brainstem (*n* = 3, 9.7%), prosencephalon and cerebellum (*n* = 3, 9.7%), cerebellum only (*n* = 2, 6.5%), and prosencephalon, cerebellum and brainstem (*n* = 1, 3.2%). The median maximum diameter was 13 mm (range, 2–40). The imaging characteristics are listed in Tables 2 and 3.

**TABLE 2:** Main computed tomography findings observed in a cohort of dogs with brain metastasis, stratified by primary tumour histotype.

	Haemangiosarcoma (*n* = 20)	Carcinoma (*n* = 16)	Melanoma (*n* = 3)
Margins			
Well‐defined	12 (60.0%)	10 (62.5%)	2 (66.7%)
Poorly demarcated	8 (40.0%)	6 (37.5%)	1 (33.3%)
Contrast enhancement			
Mild	0 (0.0%)	0 (0%)	0 (0.0%)
Moderate	5 (25.0%)	1 (6.2%)	1 (33.3%)
Intense	15 (75.0%)	15 (93.8%)	2 (66.7%)
Type of enhancement			
Homogeneous	9 (45.0%)	9 (56.2%)	2 (66.7%)
Heterogeneous	2 (10.0%)	0 (0.0%)	0 (0.0%)
Ring	9 (45.0%)	7 (43.8%)	1 (33.3%)
Mass effect	10 (50.0%)	15 (93.8%)	1 (33.3%)
Perilesional oedema	4 (20.0%)	11 (68.8%)	0 (0.0%)

**TABLE 3:** Main magnetic resonance imaging findings observed in a cohort of dogs with brain metastasis, stratified by primary tumour histotype.

	Haemangiosarcoma (*n *= 5)	Carcinoma (*n *= 0)	Melanoma (*n *= 1)
Margins			
Well‐defined	4 (80.0%)	—	1 (100.0%)
Poorly demarcated	1 (20.0%)	—	0 (0.0%)
Contrast enhancement			
Mild	0 (0.0%)	—	0 (0.0%)
Moderate	2 (40.0%)	—	0 (0.0%)
Intense	3 (60.0%)	—	1 (100.0%)
Type of enhancement			
Homogeneous	5 (100.0%)	—	0 (0.0%)
Heterogeneous	0 (0.0%)	—	0 (0.0%)
Ring	0 (0.0%)	—	1 (100.0%)
Mass effect	4 (100.0%)	—	1 (100.0%)
Perilesional oedema	4 (100.0%)	—	1 (100.0%)

Information regarding the presence of neurologic signs was available for 23 (74.2%) dogs at the time of BM diagnosis. Neurologic signs were documented in 17 (73.9%) dogs. Forebrain clinical signs were described in eight dogs, including seven patients with only epileptic seizures and one case with left pleurothotonus and bilateral reduction in menace response. Obtundation, suggestive of brainstem involvement or increased intracranial pressure, was observed in seven dogs. In two dogs, it was associated with mild to moderate tetraparesis. Six (26.1%) dogs had no neurologic signs. Four out of the 26 dogs with synchronous BM underwent antitumoural treatment, consisting of metronomic therapy in 3 dogs and splenectomy due to a ruptured tumour in 1 dog. Two dogs received palliative care.

The five dogs with metachronous BM had also received treatment at the time of their primary cancer diagnosis, consisting of surgery and doxorubicin (*n* = 3), surgery and toceranib (*n* = 1), or surgery only (*n* = 1). At the onset of BM, none of them received additional treatment. All dogs died due to tumour‐related causes with a median SPBM of 1 day (range, 1–131).

### 3.3 Dogs With Carcinoma

Among the 16 dogs with carcinoma, the presence of BM was documented through TBCT in all cases. The following primary sites were described: mammary gland (*n* = 4, 25%), lung (*n* = 4, 25%), and one (6.3%) each of nasal cavity, skin, liver, anal sac, prostate, kidney, digit and thyroid gland.

Eight (50%) dogs had synchronous BM, and the remaining eight (50%) dogs developed metachronous metastases after a median time of 399 days (range, 53–657). Metachronous metastases occurred in 3 (75%) dogs with mammary carcinoma, whereas all (100%) lung cancer cases had synchronous metastases.

At the time of BM diagnosis, seven (43.8%) dogs had nodal and distant metastases. Four (25%) dogs had only distant metastases, and three (18.8%) dogs only had nodal metastases.

A total of 11 (68.8%) dogs had a single brain lesion, while the remaining 5 (31.3) had a median number of 5 lesions (range, 2–7). Lesions were located in the prosencephalon (*n* = 13, 81.3%) and prosencephalon and cerebellum (*n* = 3, 18.7%). The median maximum diameter was 15 mm (range, 8–26). The imaging characteristics are listed in Table 2.

Information regarding the presence of neurologic signs was available for 13 (81.3%) dogs at the time of BM diagnosis. Neurologic signs were documented in 10 (76.9%) dogs. Forebrain clinical signs were observed in eight dogs, including three dogs with only epileptic seizures, disorientation in three cases, and epileptic seizures associated with compulsive aimless gait and disorientation in two patients. One dog exhibited a hypermetric gait followed by progressive tetraparesis, indicative of cerebellar involvement, while one dog showed obtundation. Three (23.1%) dogs showed no neurologic signs. Only one of the eight dogs with synchronous metastases received treatment consisting of toceranib.

Seven out of the eight dogs diagnosed with metachronous BM had received treatment at the time of their primary cancer diagnosis. Treatments included surgery alone (*n* = 3), surgery and metronomic therapy (*n* = 1), surgery and toceranib (*n* = 1), radiation therapy and toceranib (*n* = 1) and radiation therapy alone (*n* = 1). At the onset of BM, two dogs continued to receive toceranib and metronomic therapy, respectively, and two dogs received palliative care. All dogs died due to tumour‐related causes with a median SPBM of 7 days (range, 1–137).

### 3.4 Dogs With Melanoma

Among the seven dogs included in this cohort, five (71.4%) had oral melanoma, and two (28.6%) had digit melanoma. The presence of BM was documented through TBCT in three (42.9%) dogs, MRI in one (14.3%), and necropsy in three (42.9%) dogs. The necropsy was also performed on one of the dogs diagnosed via TBCT, histologically confirming the diagnosis.

All dogs developed metachronous metastases after a median time of 339 days (range, 247–1021) from the initial primary cancer diagnosis. At the time of BM diagnosis, four (57.1%) dogs had only distant metastases and one (14.3%) dog had distant and nodal metastases.

All dogs had a single lesion, located in the prosencephalon in six (85.7%) and in the cerebellum in one (14.3%); the median maximum diameter was 11 mm (range, 6–30). The imaging characteristics are listed in Tables 2 and 3.

At the time of the BM diagnosis, neurologic signs were documented in six (85.7%) dogs. These signs included forebrain signs (*n* = 4), such as compulsive aimless gait and disorientation in two dogs and epileptic seizures in the other two. One dog exhibited reduced menace response in the left eye, while another showed obtundation. One (14.3%) dog showed no neurologic signs. All dogs received treatment for the primary cancer, consisting of surgery (*n* = 4) or radiation therapy (*n* = 3); six dogs were also treated with chemotherapy. At the onset of BM, four (57.1%) dogs received treatment: symptomatic care in two cases and investigational immunotherapy in the other two. All dogs died due to tumour‐related causes with a median SPBM of 21 days (range, 1–255).

### 3.5 Dogs With Sarcoma Other Than Haemangiosarcoma

Among these four dogs, three had an undifferentiated sarcoma involving, respectively, the subcutis, gastric wall and scapula, whereas one dog had a splenic stromal sarcoma. The presence of BM was documented through TBCT in two (50%) dogs at the time of initial cancer staging, MRI in one (25%) at the onset of neurologic signs, and necropsy in one (25%) dog. Three (75%) dogs had synchronous BM, while the remaining dog with gastric wall sarcoma developed metachronous metastases after 461 days. At the time of the BM diagnosis, three (75%) dogs had distant and nodal metastases, and one (25%) only had distant metastases.

One (25%) dog had a single brain lesion, and the other three (75%) had a median number of 5 BM (range, 2–10). In all dogs, BM were located in the prosencephalon and had a median maximum diameter of 7 mm (range, 2–15). Based on TBCT, BM were well‐defined in both dogs, had intense contrast enhancement, and exhibited ring enhancement. In no case was there evidence of mass effect or perilesional oedema. The single lesion in the dog undergoing MRI was ill‐defined, had intense contrast enhancement, and showed homogeneous enhancement. There was no evidence of mass effect or perilesional oedema. At the time of the BM diagnosis, neurologic signs were documented in one (25%) dog only, presenting with obtundation. The three dogs with synchronous BM received no treatment. The dog with gastric wall sarcoma initially received chemotherapy (carboplatin) but did not undergo additional treatment at the onset of BM. All dogs were dead due to tumour‐related causes with a median SPBM of 1 day (range, 1–49).

### 3.6 Outcome and Statistical Analysis

At data analysis closure, all dogs had died. In 38 (65.5%) dogs, the cause of death was attributable to BM. The overall median SPBM was 3 days (range, 1–255). The 3‐ and 6‐month survival rates were 8.6% and 1.7%, respectively. Among the 16 dogs receiving treatment for their BM, either antitumoural or symptomatic, 5 (31.3%) had a clinical benefit, defined as an improved quality of life according to the owners. None of the investigated variables was significantly associated with a shorter SPBM.

Dogs with primary cancer locations in the heart, lung and bone (OR: 5.1; 95% CI, 1.0–25.6; *p* = 0.046), dogs with haemangiosarcoma (OR: 7.6; 95% CI, 2.2–25.8; *p* = 0.001) and those with distant metastases at presentation (OR: 16; 95% CI, 4.2–60.9; *p *< 0.001) had an increased likelihood of developing synchronous BM.

Dogs with haemangiosarcoma were more likely to have multiple BM (OR: 4.2; 95% CI, 1.3–13.3; *p* = 0.015). No other significant relationships were observed between tumour histotype and BM characteristics. Dogs with carcinoma were more likely to have nodal metastases both at first presentation (OR: 4.7; 95% CI, 1.3–17.3; *p* = 0.021) and at BM diagnosis (OR: 6.1; 95% CI, 1.7–21.4; *p* = 0.005).

## 4 Discussion

Brain metastases develop following the haematogenous spread of cells from a primary tumour to the brain microvasculature. This complex process unfolds over several stages and requires cancer cells with specific genetic traits that enable them to breach the blood‐brain barrier and adapt to the brain's unique microenvironment (Liotta and Kohn 2003). Cancer cells invade blood vessels by degrading the extracellular matrix and enter circulation, where they form protective clusters or suppress immune responses to survive hostile conditions. Upon reaching the brain microvasculature, they arrest capillaries and extravasate by adhering to the endothelium and disrupting the blood‐brain barrier through enzymatic degradation or co‐opting leukocyte pathways. Once in the brain, metastatic cells exploit local growth factors and modulate glial and immune responses to establish a supportive, tumour‐permissive microenvironment. To sustain growth, they induce angiogenesis, forming new blood vessels for oxygen and nutrients (Izadi et al. 2025).

In this study, we analysed 58 dogs with histologically confirmed primary solid tumours and BM documented via advanced imaging (TBCT/MRI) or necropsy findings. Strict inclusion criteria were applied to minimize potential misclassification and enhance diagnostic accuracy.

In line with previous literature, haemangiosarcoma was the most prevalent histotype, accounting for 53% of cases (Song et al. 2013; Snyder et al. 2006). Haemangiosarcoma, originating from pluripotent bone marrow progenitor cells, quickly reaches the circulatory system, which makes it prone to metastasis in virtually any organ, including the brain. Among the 31 haemangiosarcomas, 11 (35.5%) originated from the spleen and 8 (25.8%) from the heart.

We also found that dogs with haemangiosarcoma were more likely to develop synchronous BM compared to other malignancies; additionally, BM were more likely to be multiple. It is likely that haemangiosarcoma, due to its origin, can hijack pre‐existing blood vessels through the mechanism of vascular co‐option, resulting in diffuse brain colonization after having crossed the blood‐brain barrier (García‐Gómez and Valiente 2020). Vascular co‐option occurs when cancer cells integrate into the architecture of pre‐existing blood vessels instead of inducing the formation of new ones. This process is particularly advantageous for tumours like haemangiosarcoma, as it allows for rapid establishment in well‐vascularized tissues such as the brain. By adhering to and migrating along the endothelial lining of host vessels, tumour cells can gain access to nutrients and oxygen necessary for survival and proliferation without the time and resource‐intensive process of angiogenesis (Harris et al. 2024). These results suggest the need for increased awareness regarding the possibility of intracranial spread in dogs with haemangiosarcoma.

Carcinoma (27.6%) and melanoma (12.1%) were also common types of cancer presenting with BM, in line with previously reported data (Song et al. 2013). Among carcinomas, four (25.0%) dogs had a primary mammary malignancy, and four (25.0%) additional dogs had a primary lung tumour. The finding in the lung is noteworthy, as it has not been reported previously and because it mirrors what has been described in humans (Siegel et al. 2020).

Overall, in the current study, 63.8% of dogs had synchronous BM. However, this result should be interpreted with caution, as it may reflect the greater likelihood of performing advanced diagnostics and/or necropsy at the time of initial presentation rather than the true prevalence of synchronous BM. Notably, all dogs with melanoma in this study developed metachronous BM, compared with 8 out of 16 (50%) cases of carcinoma and 5 out of 31 (16.1%) cases of haemangiosarcoma. Within the carcinoma group, 75% of mammary cancers had metachronous BM, while 100% of lung carcinomas had synchronous BM. This pattern may, in part, be due to how these tumours are typically detected: mammary cancer and melanoma are often identified during physical examination, allowing for timely treatment. As survival rates improve for dogs with treated malignancies, the likelihood of BM development over time may also increase. In contrast, malignancies originating from internal organs, such as lung carcinoma and haemangiosarcoma, are frequently diagnosed at advanced stages, potentially explaining the higher incidence of synchronous BM in these dogs.

The prosencephalon was more frequently involved, regardless of the histotype, which is in line with previous reports (Song et al. 2013; Snyder et al. 2006). In human medicine, the distribution of BM varies depending on the type of primary cancer, suggesting that different tumour histotypes may follow distinct metastatic pathways (Cardinal et al. 2021). This pattern of dissemination has been used to help predict the primary tumour site in human patients. Unfortunately, in our study, we were unable to establish any correlation between tumour histotype and the specific brain region affected, likely due to the limited sample size, which restricted our ability to detect potential associations. However, it remains possible that similar histotype‐specific metastatic patterns exist in dogs, warranting further investigation in larger studies.

Another noteworthy finding is that 30 (51.7%) dogs had a single BM, consistent with human data (Nayak et al. 2012). Additionally, all (100%) dogs with melanoma had a solitary lesion. The diagnosis was obtained via TBCT in 23 (76.7%) dogs, necropsy in 4 (13.3%), and through MRI in 3 (10%). Although we cannot exclude that the number of BM depends on the specific cancer type, as some malignancies are more likely to metastasize to the brain in a multifocal pattern rather than forming solitary lesions, it is also possible that the number of BM was underestimated if CT was used as the imaging technique. In the current study, 36.6% of patients undergoing CT showed multiple lesions, while MRI conducted on seven dogs revealed multiple lesions in 71.4% of them. This difference may underscore the greater sensitivity of MRI in detecting BM. Similarly, in human medicine, the limitations of CT in detecting the number of BM are well recognized. Approximately 19% of patients with a solitary metastasis on contrast‐enhanced CT are found to have multiple metastases on a contrast‐enhanced MRI (Schellinger et al. 1999). The number of BM may also have been underestimated in cases diagnosed through necropsy alone, particularly if only gross examination was performed without extensive histopathological sampling.

While the majority of dogs showed neurologic signs, 21% of dogs had asymptomatic BM, suggesting that intracranial pathology may be underreported in historical data. Interestingly, all asymptomatic dogs had forebrain metastases. This intriguing finding reflects the ability of the canine forebrain to ‘mask’ brain involvement and produce obvious clinical signs only in the most severe cases. These results suggest a potential role for surveillance brain imaging in dogs with solid cancers displaying neurotropism. However, it is currently unclear whether detecting occult BM provides a survival benefit by enabling treatment of the brain.

In our patient group, the presence of BM was associated with multiple distant and/or nodal metastases in 89.7% of cases, and BM was the sole site of tumour spread in only 10.3% of dogs. This information is crucial for clinicians and radiologists and supports the use of combined brain MRI and TBCT for comprehensive staging if BM are suspected.

There is no established treatment of choice against BM reported in veterinary medicine. Here, over 70% of dogs did not receive any treatment, whereas 15.5% of dogs underwent various medical antitumoural treatments. Although survival was not extended, some clinical benefit was reported in a small subset of treated dogs. Due to the small sample size and the heterogeneity of treatments, no definitive conclusions can be drawn.

The prognosis was poor, and the majority of dogs (65.5%) died due to BM, indicating that brain spread probably represents a final event in the course of the disease. The median survival after BM diagnosis was 3 days, and the 6‐month survival rate was 1.7%. The reason can be attributed to the severe clinical condition, the lack of effective therapies and/or the poor control of extracranial disease, considering that nearly all dogs had distant metastases at the time of diagnosis of BM. It is important to note, however, that this figure includes both spontaneous deaths and euthanasia. Therefore, the high proportion of BM‐related deaths may be influenced by a decision‐making bias: many owners may opt for euthanasia upon the onset of neurologic signs or following the diagnosis of BM based on anticipated deterioration and limited treatment options. This potential bias should be considered when interpreting the data.

This study carries all the limitations and biases of a retrospective design. The number of dogs was small, given the rarity of the diagnosis, which limited the statistical analysis. Not all dogs underwent specialized neurologic examination, which may have led to an underestimation of mild neurologic deficits, particularly in dogs that were not initially evaluated at a neurology referral clinic. However, all dogs underwent a detailed anamnesis to assess for seizures or other behavioural abnormalities, as well as a thorough physical examination that included key neurologic parameters such as mental status and gait assessment. These parameters are frequently altered in patients with intracranial pathology and provide valuable clinical insight, even in the absence of a complete neurologic evaluation. Similarly, not all dogs underwent standardized diagnostic work‐up. The majority were evaluated using CT, with only seven undergoing MRI. Additionally, only a small subset of dogs received antitumoural therapy, which was exclusively pharmacologic. Therefore, it is not even possible to speculate on whether local therapies, such as surgery and radiotherapy, commonly used in human patients with BM, may have any efficacy in dogs. Additionally, only 14 dogs underwent necropsy, definitively confirming the presence of metastases and their origin. Unfortunately, ante‐mortem biopsies are rarely proposed, both due to technical difficulties and because it would be unethical in fragile patients with a short life expectancy.

Due to the study design and the fact that one centre is referred for neurology cases only, it was not possible to calculate the prevalence by comparing demographics with dogs without BM. Therefore, it remains currently unknown how frequently dogs with haemangiosarcoma and carcinoma exhibit or develop BM.

Lastly, due to the retrospective nature of the study and variability in MRI unit types and magnet strengths, MRI protocols were not standardized. As a result, certain specialized sequences, such as susceptibility‐weighted imaging, were not consistently included. These sequences could have been useful in better characterizing the observed lesions (Delmaire et al. 2015).

In conclusion, we found that haemangiosarcoma, carcinoma and melanoma accounted for the majority of all BM. Although this is largely due to the prevalence of these tumours, it also reflects the organotropism of certain malignancies toward the brain. BM were more often synchronous and symptomatic; nevertheless, a subset of dogs exhibited no neurologic signs. MRI more frequently revealed multiple BM, while CT demonstrated extracranial metastatic spread, supporting the diagnosis of BM. The prognosis was poor, regardless of the primary cancer type. Collaborative multi‐institutional studies, including cooperation between the oncologist, the neurologist and the radiologist, are warranted to improve the knowledge and early detection of BM, which would lead to the development of efficacious treatments.

## Author Contributions

**Samuel Okonji**: acquisition of data, analysis and interpretation of data, writing, review, and/or revision of the manuscript. **Federica Rossi**: acquisition of data, writing, review, and/or revision of the manuscript. **Silvia Sabattini**: laboratory investigation, analysis and interpretation of data, writing, review, and/or revision of the manuscript. **Massimo Baroni**: acquisition of data, writing, review, and/or revision of the manuscript. **Federica Poli**: acquisition of data, writing, review, and/or revision of the manuscript. **Riccardo Zaccone**: writing, review, and/or revision of the manuscript. **Simone Perfetti**: acquisition of data. **Gualtiero Gandini**: analysis and interpretation of data, writing, review, and/or revision of the manuscript. **Laura Marconato**: development of methodology, acquisition of data, analysis and interpretation of data. writing, review, and/or revision of the manuscript, study supervision.

## Ethics Statement

In compliance with local legislation, ethical approval was not required for this study. Dogs were treated according to the current standards. All owners signed a written informed consent.

## Conflicts of Interest

The authors declare no conflicts of interest.

### Peer Review

The peer review history for this article is available at https://www.webofscience.com/api/gateway/wos/peer‐review/10.1002/vms3.70441.

## Data Availability

The data that support the findings of this study are available from the corresponding author upon reasonable request.
